# Comparative study of subcutaneous, intramuscular, and oral administration of bovine pathogenic *Escherichia coli* bacterial ghost vaccine in mice

**DOI:** 10.3389/fimmu.2022.1008131

**Published:** 2022-11-14

**Authors:** Jing Mu, Lei Lei, Yingce Zheng, Ding Li, Jie Li, Yunwei Fu, Guanbo Wang, Yun Liu

**Affiliations:** ^1^ Key Laboratory of Comparative Medicine, Department of Veterinary Surgery, College of Veterinary Medicine, Northeast Agricultural University, Harbin, China; ^2^ College of Life Science, Northeast Agricultural University, Harbin, China; ^3^ Veterinary Medical Teaching Hospital, Northeast Agricultural University, Harbin, China; ^4^ Wellcome-Wolfson Institute for Experimental Medicine, Queen’s University Belfast, Belfast, United Kingdom

**Keywords:** bacterial ghosts, *Escherichia coli*, pBV220 vector, lysis cassette, humoral immunity

## Abstract

*Escherichia coli* is one of the most common bacterial pathogens in cattle. Prophylactic vaccines are considered promising strategies with the potential to reduce the incidence of colibacillosis. Some studies suggested that bacterial ghosts may serve as a novel approach for preventing bacterial infections. However, the roles of administration route on vaccine immunogenicity and efficacy have not been investigated. In this study, the efficacy of vaccination *via* different immune routes in generating humoral and cellular immune response was compared through subcutaneous (SC), intramuscular (IM), and oral (O) administration in female BALB/c mice with bacterial ghosts prepared using wild type *Escherichia coli* isolates CE9, while phosphate buffer saline (PBS) and inactivated vaccines containing aluminum adjuvants (Killed) were used as control. Our results showed that the plasmid pBV220-*E-*aa-*SNA* containing *E. coli* was efficiently cleaved at 42°C with 94.8% positive ratio as assessed by colony counts. Transmission electron microscopy (TEM) confirmed bacteria retained intact surface structure while devoid of cytoplasmic component. We found that total IgG titers in killed, IM and SC groups showed significant increase on 7, 14, 21 and 28 days post-immunization. The IgA level of the IM group was higher than that of all other groups on the 28th day. Meanwhile, four experimental groups showed a significant difference in IgA levels compared with PBS control. In the IM group, an increase in the relative percentages of CD3+CD4+ T cells was accompanied by an increase in the relative percentages of splenic CD3+CD8+ T cells. In comparison with the inactivated vaccine, intramuscular CE9 ghosts immunization elicited higher levels of IL-1β, IL-2, IL-6 and IL-12. Subcutaneous and intramuscular immunizations were significantly associated with improved survival in comparison with oral route, traditional vaccine and the control. Pathologic assessment revealed that less severe tissue damage and inflammation were found in lung, kidney, and intestine of IM group compared with other groups. The results above demonstrate that immunization of *Escherichia coli* CE9 ghosts *via* intramuscular injection elicits a more robust antigen-specific immune response in mice to prevent the *Escherichia coli* infection.

## Introduction

Escherichia coli (E.coli), a gram-negative bacterium in the family Enterobacteriaceae, induces multi-species bacterial diseases and is widespread all over the world (1). Infections with *E.coli* are common in avian, humans and other mammals, posing a threat to food safety and public health. Infected animals and contaminated water can severely affect human health, causing neonatal sepsis, bacterial meningitis, diarrhoeal disease, and urinary tract infection (2–4). Compared with humans, *E. coli* infection in animals is often limited to clinical monitoring result in recessive infection state, which exhibit long-lasting consequences for the environment (5, 6).

Bovine colibacillosis represents an ongoing and major challenge to the livestock industry due to the role of *E.coli* as the commonest bacteriological pathogen in several growth and development stages with substantial economic consequences. Although most *E. coli*, which is found ubiquitously in the environment, are not pathogenic for animals in healthy conditions, postpartum cows and newborn calves are more susceptible to bacterial infections due to immunocompromised state. In calves, enterotoxigenic *E. coli* infection can cause severe diarrhea; in dry period, intramammary *E. coli* infection causes mastitis, resulting in decreased milk production and poor milk quality (7, 8). Infection during each stage of pregnancy and labor may lead to uterine inflammation and even fatal bacteremia and septicemia. Currently, antibiotics are still the mainstay of treatment for *E. coli*-caused infections. For example, on some cattle farms, antibiotics were added into the daily feed to prevent colibacillosis. Meanwhile, use and abuse of antibiotics led to antibiotics resistance getting more and more serious over time (9, 10). A study in the USA reported that resistance to extended-spectrum cephalosporins increased from 5.46% to 12.97% and fluoroquinolones resistance reached 28.22% during the 8-year study period (11). In England, there were 616 multidrug-resistant (MDR) out of 1434 *E. coli* isolates from Oxfordshire (12). In a recent study, a bad trend was discovered that may be causing more severe concerns about antibiotic resistance. It was reported that even without using quinolones, using other antibiotics also contributed to acquiring quinolone resistance (13). Instead of treatment with antibiotics, vaccines provide long-lasting protection from bacterial infections for animals through periodic immunization in the livestock industry. The diversity of serotypes and cross-protection between them pose a significant challenge in vaccine research and development.

As the main immunological feature of *E. coli* organisms, the bacteriophage antigen, O antigen, is still used as the main basis for *E. col*i serotype classification and also influences its virulence and pathogenicity (14). Enormous structural diversity of O antigens lies in the sugar composition and the linkages between monosaccharides (15). Currently, as many as 185 O antigens are known. The poor cross-protection between serotypes has been demonstrated in practice, which means that it is difficult to cover multiple serotypes of *E. coli* infection with immunization against a few antigens (16). From the perspective of pathogenicity, immunization against colonization factors and adhesins in specific pathogenic *E. coli* can prevent their infection (14). However, virulence factors are often transferred horizontally between bacteria *via* plasmids, islands, phages, etc., which is making widespread prevention of *E. coli* by immunization difficult. Even though, attempts using new strategies for vaccine development have shown promising results (17, 18).

During the past several decades, novel vaccines have gained increasing attention to achieve better immune protection. Among them, bacterial ghosts (BGs) have been attracting significant interest among researchers due to their potent adjuvant effects and favorable immunologic effects (19, 20). BGs are cell envelopes originated from gram-negative bacteria losing cytoplasmic contents but maintaining morphology and surface structure such as lipopolysaccharides (LPS), outer membrane proteins, adhesins, etc. The formation mechanism for BGs is that channels in the bacterial cell membrane by genetic engineering methods or chemical approaches leads to cytoplasmic contents flowing to the surrounding medium, sequentially leaving an empty bacterial shell without nucleic acid. BGs are also considered as an intruder by the immunity system because of antigenic determinant on the surface of bacteria cells, which can be recognized by macrophages and in turn eliciting systemic humoral and cellular immune responses. As vaccine, BGs can trigger elevated levels of antibodies (21); as an adjuvant, they have proven to improve the protective efficacy of *Chlamydia abortus* and *Newcastle disease virus* vaccines (22–24).

Currently, genetic engineering is the most common method used to produce BGs. Many studies have shown that protein E, which is translated from the lysis gene *E* of the ΦX174 bacteriophage, has a lytic function on bacteria. However, the mechanism of the effect has not been fully elucidated. Initial recognition of the *E* gene cleavage function was based largely on morphology. The cell envelope breaks down more gradually in two distinct stages: the cell wall rolls back, then the plasma membrane disintegrates. However, as the authors state, it is not possible to draw any detailed conclusions regarding the mechanism of breakdown of the cell envelope at a macro-molecular level in either case from micrographs (25). In the following study, other groups attributed the function of the *E* gene to the activation of unspecified autolytic functions or the formation of polymeric “transmembrane tunnels” that opened the cytoplasm directly to the medium based on physiological and scanning-EM studies (26). In the two-stage model, the assumption of “transmembrane tunnels” was further demonstrated and widely accepted: Protein E induces the generation of transmembrane channels (40~200nm) *via* (a) protein E integration into the inner membrane; (b) protein conformational change transferring the C-terminus across the inner membrane, and(c)fusion of the inner and outer membranes. Cytoplasmic contents such as nucleic acid, ribosomes, etc. flowed out *via* these transmembrane channels because of osmotic pressure between the internal and external environments of the cell, resulting in a complete bacterial envelope (27). Starting with gene *E*-induced *vibrio cholerae* ghosts by Werner Lubitz in 1994 (28), constant modification of gene *E* as well as vectors enhances cleavage efficiency in some bacteria (29). Researches have shown that fusion genes of mutant gene *E* and *staphylococcal nuclease A* (*SNA*) gene not only induce the genetic inactivation of *E.coli* cultures efficiently but also prevent horizontal gene transfer which contribute to the propagation of antibiotic resistance (30). Thus, in this study, the tandem two genes (*E*+*SNA*) were utilized for bacteria BG production.

As a novel vaccine strategy, it is important to optimize the immune conditions for BGs to achieve a better protective effect. Considering the special structure and infection routes, BGs can be administered *via* subcutaneous (SC), intramuscular (IM), intraperitoneal (IP), oral (O), respiratory (RP), rectal (R), etc. (31, 32). *Salmonella enterica* ghosts robustly stimulate humoral and cell-mediated immune responses in chickens *via* the intramuscular, subcutaneous, and oral routes. No significant differences in immune markers were found between immune routes, except for significantly higher IgA levels in the oral group compared to SC and IM groups (33). *Acinetobacter* ghosts have also been shown to be effective by leukocyte counts, assays of immune cell activity, and phagocytosis when administered subcutaneously, intramuscularly, intraperitoneally, and orally, but the differences between the various immunization routes remain unknown (34). In addition, as a novel vaccine, the differences in immunization effect of BGs compared with traditional inactivated vaccine are of particular interest. Currently, there are no relevant studies about bovine pathogenic *E. coli* ghosts. Therefore, in this study, we evaluated the immune effects of traditional inactive vaccines with aluminium adjuvants *via* IM and *E. coli* ghosts through subcutaneous (SC), intramuscular (IM), and oral (O) to contribute to the application of BGs as a novel vaccine candidate in the future.

## Results

### Construction of lysis plasmid containing gene *E* for generation of *E.coli* CE9 ghosts

**Figure 1:**
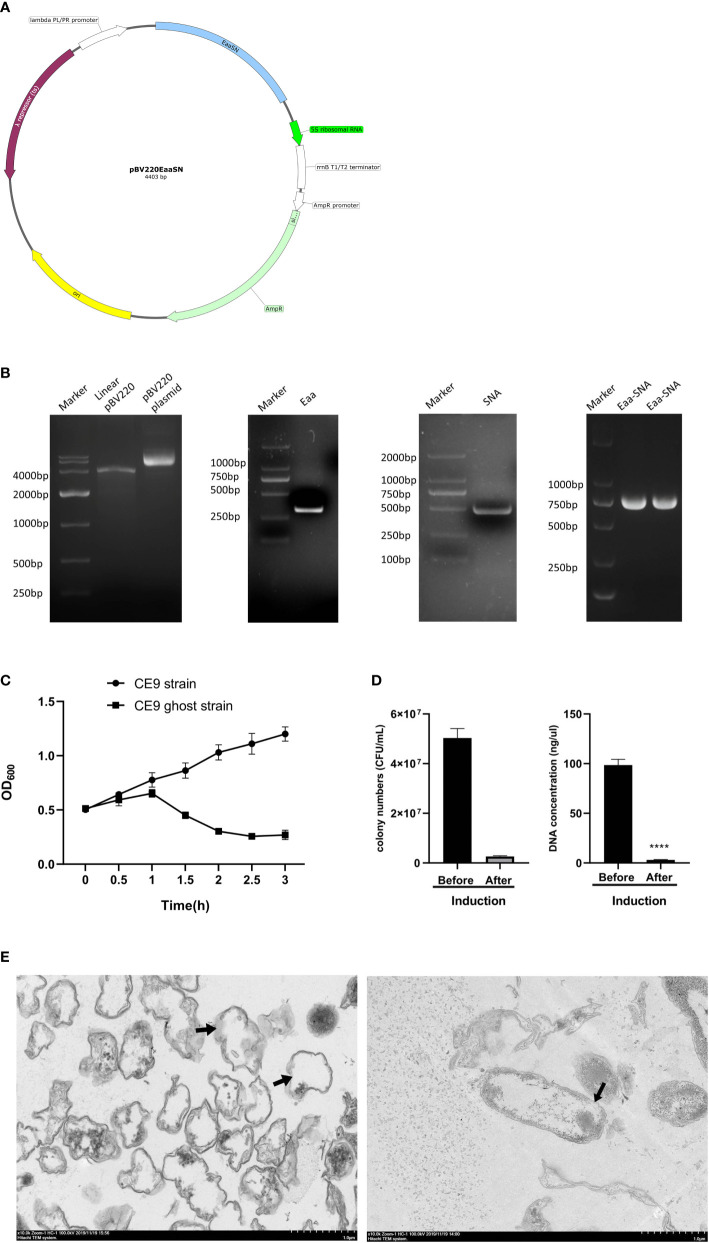
Construction of the lysis plasmid and identification of bacterial ghosts (BGs) quality. **(A)** The lysis plasmid map showing the location of elements with different colors. **(B)** PCR identification of E-lysis gene, SNA gene, and tandem fragments E-aa-SNA. **(C)**. The growth of CE9 strain and CE9 ghost strain (containing lysis plasmid) culturing at 42°C. **(D)** The lysis efficiency was indicated by the number of clones and DNA concentrations before and after lysis. **(E)** Transmission electron micrographs of the BGs. ****p<0.0001.

The plasmid map demonstrates the structure of the lysis plasmid (**Figure 1A**). The pBV220 contains the *PL* promoter and the *cIts857* regulatory gene *cI* encoding a temperature-sensitive cI protein that is repressive to the promoter and therefore can be used to regulate the transcription of the *Eaa-SNA* inserted by temperature. A strong transcriptional terminator prevents “read-through” and contributes to the stability of the plasmid-host system. Identification of each element by gel electrophoresis of each element (**Figure 1B**) The OD_600_ of the CE9 ghost strain decreased until reaching a minimum at 2.5h, while strain CE9 increased continuously (**Figure 1C**). The results of bacterial colony counting before and after induction are 5×10^7^ CFU/mL and 2.6×10^6^ CFU/mL, respectively. Therefore, the cleavage efficiency was 94.8%. In addition, the DNA concentrations of pre-induction and post-induction were 98.6 ng/μL and 3.7 ng/μL (**Figure 1D**). Inspection with electron microscopy suggested that most of the contents of the bacteria leaked out, the structure of bacteria essentially intact, empty inside, and transmembrane pores have been observed. The appearance of wrinkles on the bacterial surface may be associated with the freeze-dried process and loss of its contents (**Figure 1E**).

### The BGs were sterilized and nontoxic to mice

No colony growth was observed on plates coated with resuspensions of lyophilized CE9 BGs, suggesting that the remaining viable bacteria had died during the lyophilization. In the safety test, no abnormal behavior such as lethargy, decreased appetite, diarrhea, or weight loss was observed in the mice.

### The IgG and IgA levels induced by *E.coli* CE9 ghosts in mice serum

**Figure 2:**
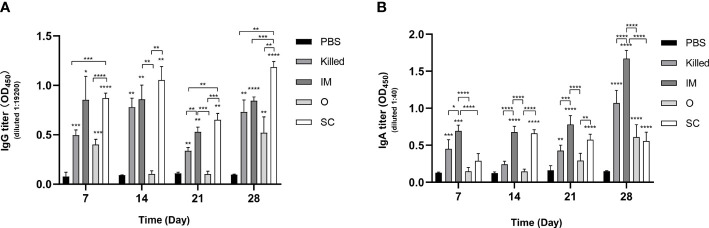
Specific IgG and IgA antibodies in mice immunized by E.coli CE9 ghosts. Specific antibody IgG levels in serum **(A)** and IgA level in intestinal lavage fluid **(B)** at 7, 14, 21 and 28 days after immunization. Significance was analyzed using 2-way ANOVA and Tukey’s multiple comparisons test. *p<0.05,**p<0.01,***p<0.001, ****p<0.0001.

IgG antibodies in serum and IgA antibodies in intestinal lavage fluid were detected by ELISA at 7, 14, 21 and 28 days post-immunization. In addition to O group, significant improvements in IgG levels in three experimental groups (Killed, IM and SC) were also observed compared to the control group (PBS-treated mice) within the whole 28-day immunization period. The average IgG levels of the IM group and SC group were persistently higher than PBS. The SC group appeared to have a rapidly increased IgG levels and showed the highest mean antibody level among all the groups. There was no significant difference between SC and IM during the first 21 days. However, the IgG level of SC was significantly increased compared to that of the IM group on the 28th day. Surprisingly no stable and continued IgG of the O group was detected. All the experimental groups ranked by IgG level from high to low are: SC, IM, Killed, and O (**Figure 2A**).

Total IgA titers determined by ELISA indicated that the mean IgA titer of the IM group was higher than that of all other groups on days 7, 14, 21, and 28. Finally, the IgA titers of Killed, O and SC showed significant differences compared with the control group (**Figure 2B**). It is noteworthy that the IgA levels of the O group were gradually increased within the 28 days, despite showing lower IgA titers than other experimental groups.

### Cellular immune responses and spleen coefficients

**Figure 3:**
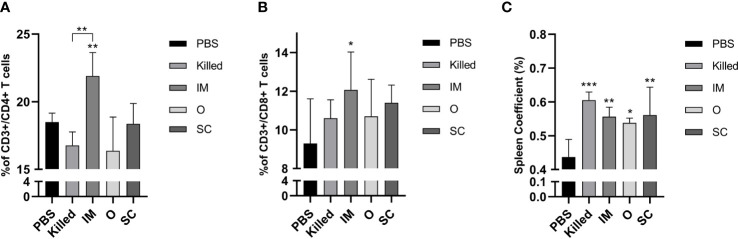
Cellular immune responses of vaccinated mice. Percentage of CD3+/CD4+ **(A)** and CD3+/CD8+ **(B) **T cells in spleen detected by flow cytometry at day 28 post-immunization. The spleen coefficient **(C)** was calculated by spleen weight (g)/mice weight (g). Significance was analyzed using 1-way ANOVA and Tukey’s multiple comparisons test. *p<0.05,**p<0.01,***p<0.001.

In order to assess the cellular immune responses induced by bacterial ghosts, CD3+/CD4+ and CD3+/CD8+ T cell populations in spleens were measured by flow cytometry. Only the percentage of CD4+ and CD8+ cells of the IM group was significantly higher than the control (**Figures 3A, B**). No notable differences were observed among the other experimental groups. Spleen size was assessed as a percentage of body weight and calculated on day 14 after secondary immunization. The result indicated that the spleen coefficients of all the experimental groups were increased compared with the control group (**Figure 3C**).

### Cytokines secretion *via* activating Th1/Th2 type T cells induced by CE9 BGs

**Figure 4:**
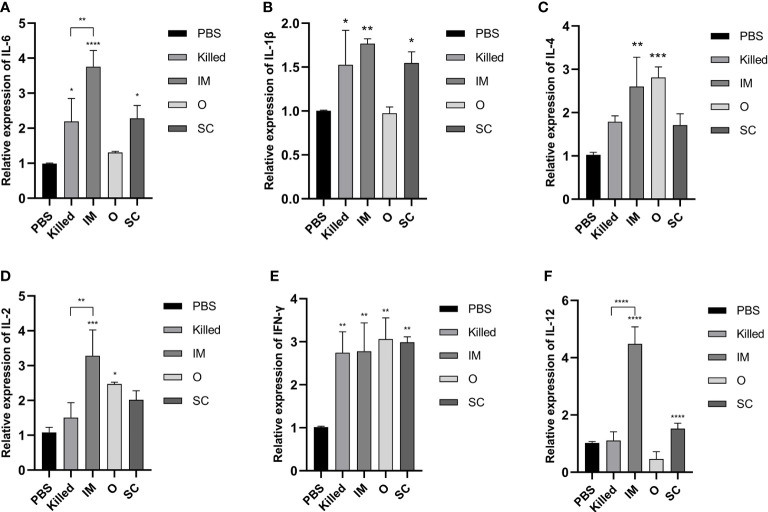
Cytokine expression was assessed by RT-qPCR normalized to GAPDH at day 28 post-immunization. All groups were compared to the PBS group. The expression of IL-6 **(A)** and IL-1β **(B)** in the Killed group, IM group, and SC group were significantly elevated. The IL-4 expression levels **(C)** in the IM and O groups were significantly higher than the other groups. The IM group had the highest IL-2 expression level **(D)** among all groups. IFN-γ titers **(E)** were significantly higher in all immunization groups. The IM group had the highest IL-12 titer **(F)** among the groups. Significance was analyzed using 1-way ANOVA and Tukey’s multiple comparisons test. *p<0.05, **p<0.01, ***p<0.001, ****p<0.0001.

To better understand T-cell activation and differentiation after BGs vaccination, cytokines gene transcription in peripheral blood at day 28 post-immunization was analyzed using RT-qPCR. IL-6 and IL-1β expression of Killed, IM and SC as well as IL-4 and IL-2 expression of IM and O, were significantly higher than in controls (**Figures 4A-D**). IFN-γ expression levels were higher in all experimental groups (**Figure 4E**). Furthermore, it was clear that the IL-12 expression of IM was much higher than all of the other groups, followed by SC (**Figure 4F**). After booster immunization, the expression of both Th1 cytokines (IL-2, IL-12) and Th2 cytokines (IL-6) of IM showed differences with those of the Killed group. The above results suggested that compared with traditional vaccines, BGs elicited a stronger cell-mediated immune response by activating Th1/Th2 type T cells.

### Protective efficacy challenged with pathogenic *E.coli* CE9 in mice

**Figure 5:**
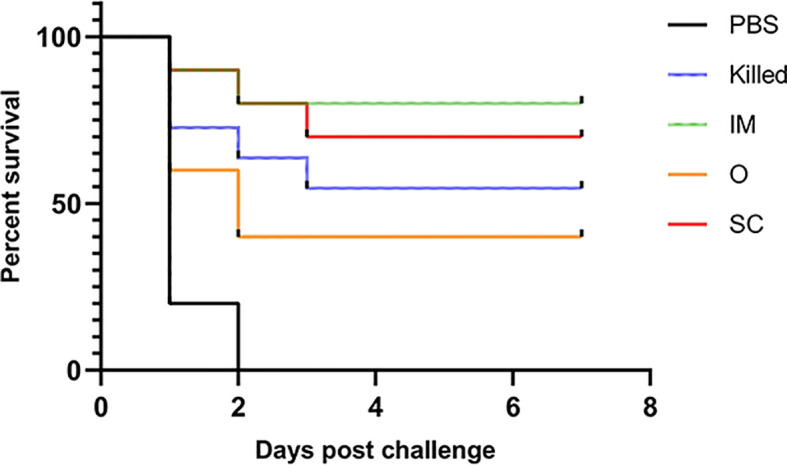
Survival rates of vaccinated mice after challenge with 5 LD_50_ CE9 strain. Except for the PBS group, all experimental groups protected some mice from the CE9 strain-induced death.

The control group, mice immunized with PBS, all died within 48 h. The protection rate of groups IM, SC, Killed and O was 80%, 70%, 60% and 40%, respectively. We found that this result was associated with specific antibody levels (**Figure 5**).

### Histopathological examination

For the control group, we selected the mice that had just died for further research because there were no survivors. The mice of all the groups were killed and organs (spleen, kidney, and lung) harvested on the 7th day after challenge. HE staining was used to observe the pathological changes in the mice organs.

**Figure 6:**
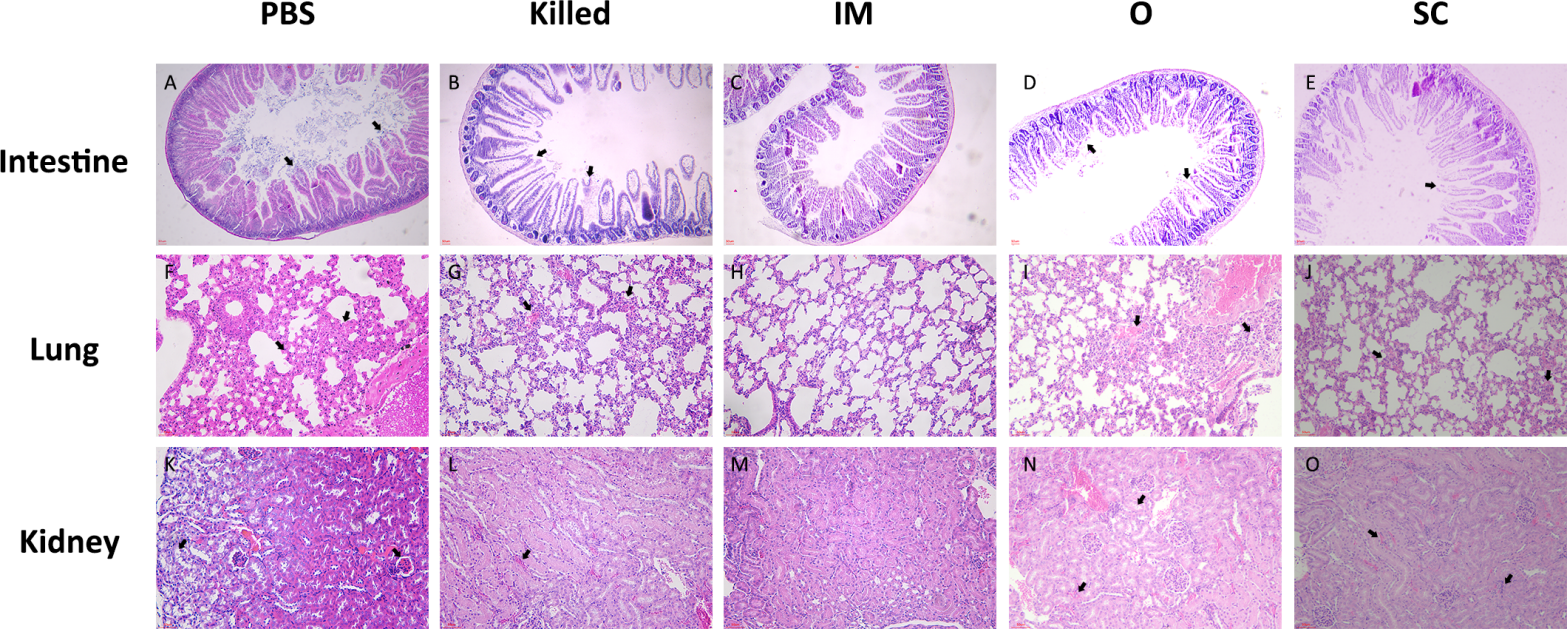
Pathological histological evaluation of intestine **(A–E)**, lung **(F–J)** and kidney **(K–O)** of mice in the PBS, Killed, IM, O and SC group at 7 days post-challenge. Intestinal villi (40×), alveoli (100×), and renal cortical structures (100×) were severely damaged in the PBS group. In the Killed group, Intestinal villi, alveoli structures were moderate damaged, while the renal cortical was mild damaged. Almost no damage appeared in the tissues of IM group. In the O group, intestinal villi showed mild injury, whereas moderate pulmonary and kidney lesions were observed. SC group showed engorged alveolar capillaries and peritubular capillaries.

In the control group, intestinal villi were seriously damaged with inflammatory cell infiltration, a large number of broken cells in the gut lumen (**Figure 6A**). In the Killed group, the structure of intestinal villi was largely intact, but a few intestinal epithelial cells shed off (**Figure 6B**). However, the intestinal villi of the IM group appeared normal, intestinal epithelial cells were basically in good order; there was minimal inflammatory cell infiltration (**Figure 6C**). The intestinal pathological findings in group O showed partial loss of gut epithelium and few villus sloughing (**Figure 6D**).SC group showed the villus structure remained relatively intact, a small number of epithelial sloughing as well as a few inflammatory cell infiltrates (**Figure 6E**).

Obvious pathological changes were observed in the lung. The alveolar epithelial cells were swollen and the alveolar walls were thickened, engorged alveolar capillaries with inflammatory cell infiltration (**Figure 6F**). In group Killed, similar but less severe pathological changes than those in control were observed (**Figure 6G**). The pulmonary structures of the IM group appeared histologically normal (**Figure 6H**). Intra-alveolar inflammatory, hemorrhage and thickened alveolar septa were observed in O group (**Figure 6I**). SC mice showed alveolar congestion and inflammatory cell infiltration in the lungs (**Figure 6J**).

The control group displayed significant renal cortex damage, including glomeruli shrunken with congestion of glomerular capillaries, tubular epithelial cell sloughing and inflammatory cell infiltration (**Figure 6K**). Despite the engorged glomerular capillaries, the Killed group showed grossly normal renal cortex structure (**Figure 6L**). Mice of IM showed mild congestion of renal blood vessels. Beyond this, no overt abnormalities were detected in the histologic appearance of the renal cortex (**Figure 6M**). In O group, peritubular capillaries congestion and hemorrhage as well as denuded epithelial surfaces were frequently observed. (**Figure 6N**) In the SC group, the glomeruli and renal tubules were structurally intact, and a small amount of peritubular capillaries congestion was observed (**Figure 6O**).

## Discussion

The expression vector for the lysis plasmid in this study was pBV220 with the temperature-sensitive regulatory system *λpL/pR-CIts857*, which initiates the translation of the fragment inserted in its polyclonal site (35). Compared to vectors using inducers such as IPTG for induction, pBV220 was more convenient. However, this plasmid also had some limitations for this research. Since positive clones containing the pBV220 plasmid exhibit ampicillin resistance, the plasmid could only be applied to those Gram-negative bacteria that are ampicillin-susceptible. Unfortunately, ampicillin resistance was extremely common in gram-negative bacteria, which would result in many bacteria not being able to prepare BGs with this plasmid. To solve this problem, we could change the screening conditions by modifying the vector. For example, the blue-white screening element on the pUC19 plasmid might sidestep the problem by inserting the entire temperature-controlled lysis cassette into the polyclonal site of pUC19 on the basis of the plasmid constructed in this experiment (31).

Although the plasmid constructed in this study could be expressed efficiently in wild-type *E. coli* CE9 strain, the lysis efficiency decreases with increasing passages, probably because of the segregational instability and structural instability (36). Therefore, unless this problem is solved, the passage times of positive clones should be minimized to achieve effective cleavage.

Complete inactivation of pathogenic *E.coli* CE9 could not be achieved by *E* gene lysis alone. Following induction, residual live bacteria needed to be further inactivated further. β-propanolactone interacts with the guanine bases of nucleic acids to inactivate bacteria, viruses, and other microorganisms, and was widely used in the preparation of virus vaccines. β-propanolactone was hydrolyzed completely to β-hydroxypropionic acid without any toxicity by reaction with water (37). β-propanolactone has been studied for the inactivation of residual bacteria after BGs induction. The remaining viable bacteria with appropriate concentrations of β-propanolactone at 60 min at 42°C could be complete inactivated. Although β-propanolactone provides good inactivation, it was not suitable for mass production due to its high price. Several studies have demonstrated that gentamicin inactivates bacteria due to its effects of methylation, inhibition of protein synthesis, and increased cell permeability (38). In the present study, no live bacteria were found in the BGs after lyophilisation, perhaps because the CE9 ghost strain did not tolerate low temperatures, so no additional inactivation reagents are needed to ensure vaccine safety. Inactivation seemed like an easy problem to solve due to the many inactivators available, but how to ensure the antigenic structure of the bacterial surface while killing residual bacteria still needs to be proven by rigorous experimental data.

For various pathogens, there were differences in the immunization routes for BGs vaccines. In this study, *E. coli* CE9, which causes diarrhea in calves, was the intestinal infection. Intramuscular and subcutaneous injections were two of the most common immunization routes, so three routes were set for the comparison of BGs immunization: oral, subcutaneous, and intramuscular. For IgA and IgG levels, all groups were immunized with a two-week interval after the first immunization, and the antibody levels were significantly increased in all experimental groups after 2 weeks of booster immunization. The SC group had higher levels of specific IgG antibodies at all times and also significantly elevated IgA levels, while the IM group had significantly higher levels of IgA antibodies at day 28 and also elicited high levels of IgG, indicating that both subcutaneous and intramuscular injections induced robust humoral immune responses. *Salmonella* ghosts in chickens have been shown to result in the highest serum-specific antibody levels by intramuscular injection compared to other immunization routes (39). These findings are in agreement with earlier reports: higher levels of IgG elicited by *Salmonella* ghosts intramuscularly than orally in chicken (40). Another study showed close antibody levels for intramuscular and subcutaneous injections of *Salmonella* ghosts, with slightly higher antibody levels in the IM group than in the SC group, which were similar to the results in this study (33). Subcutaneous immunization of BGs against *Haemophilus parvum* in swine and *Bordetella bronchiseptica* in dogs has shown good results. From these available references, subcutaneous and intramuscular immunizations were the most versatile and effective immunization routes against various BGs (31, 41, 42).

The IgG and IgA antibody levels in the oral group remained suboptimal. Taking the immune protection rate together, the antibody levels induced by the 2 immunizations were not sufficient to resist infection. Oral immunization was more convenient than injection, but it is susceptible to loss and can lead to unstable immunological effects. Previous studies have demonstrated that outer membrane vesicles (OMVs) from *E. coli* encapsulated into Zein Nanoparticles coated with a Gantrez^®^ AN–Mannosamine Polymer Conjugate elicited high levels of IgG antibodies, while oral administration of the outer membrane protein alone did not elicit an antibody response (43). In contrast, displaying CD0873 on liposomes without encapsulation elicited significantly different IgG and IgA titers from the control group after three oral immunizations (44). Oral exposure to stx+ eae+ intimin-positive *E. coli* O157: H7 as an oral vaccine twice weekly for 6 weeks would increase resistance of cattle to colonization by wild-type *E. coli* O157: H7 following challenge (45). Our findings also showed a trend of higher antibody levels for IgG and IgA with the immunization duration. These findings suggested that we may achieve better immunologic effects with oral *E. coli* ghost vaccines, but rather need to find more appropriate immune-related conditions, such as increasing the time of immunization and appropriate encapsulants.

In general, two immunizations were more effective than a single immunization. The prime-boost immunization strategy of *Salmonella Gallinarum* (SG) ghosts had an advantage over single immunization in chickens because it induces a robust immune response for optimum protection against fowl typhoid (46). *EHEC* O157:H7 BGs, on the other hand, have been shown to significantly stimulate efficient humoral and cellular immune responses and to be as protective as two immunizations (47). It illustrated that different strains, administration routes, and experimental animals need to choose different immunization protocols. In the present study, the IM group showed a significant increase in IgA-specific antibodies after the second immunization compared to the first immunization, which facilitated defense against bacterial infection. Considering that there was no significant difference in IgG antibody levels between 14 and 28 days, it remains uncertain whether the improved protective efficiency due to increased lgA levels based on mucosal immunity. Therefore, it is necessary to explore the role of booster vaccination through single and multiple immunizations of BGs *via* the intramuscular route.

It has been shown that the immunologic effects of BGs vaccines were superior to those of inactivated vaccines (21), but the adjuvants have a significant impact on the immunization effects. In this study, the inactivated vaccine containing aluminium adjuvant elicited high antibody levels and cytokine levels, but the antibody and cytokine levels and protection rate remained low relative to the intramuscular and subcutaneous injections with BGs. The local metabolism of aluminium adjuvant was found to be hard (unpublished observations). In contrast, no injection site swelling was observed after 2 intramuscular injections of BGs.

In the present experiment, single nucleated cells collected from the spleen were examined by flow cytometry for the percentage of CD4+ and CD8+ T cells, and the results indicated that CE9 ghosts elevated CD4+ and CD8+ levels by intramuscular immunization. Other routes, however, revealed no significant differences in CD4+ and CD8+ T cell levels. On the one hand, intramuscular immunity could better activate CD4+ and CD8+ T cells compared to mucosal immunity (48, 49). On the other hand, although there was no significant difference in the proportion of CD4+ or CD8 T cells compared to the control group, the elevated cytokines such as IFN-γ, IL-4, and IL-6 also reflected the cellular immunity being activated. The expression level of IL-12 was significantly higher in the IM group compared to the other groups. CD4+ T cells differentiate into regulatory T cells (Treg) in response to antigen stimulation. In the presence of TGF-β, IL-12 promotes Treg cell differentiation into Foxp3^+^ IFN-γ^+^ Treg cells as well as IFN-γ expression (50). The increase in IL-6 also contributes to the conversion of CD4+ T to Th17 in the presence of TGF-β. Th17 cells promote a direct CXCL8-dependent chemotactic effect on neutrophils, which is important for antimicrobial immunity (51). Perhaps due to insufficient sample size, it was not sensitive enough to reflect a smaller difference.

The expression of IL-1β, IL-2, IL-4, IL-6, IL-12 and IFN-γ was increased differentially in groups. IFN-γ, a signature cytokine produced by TH1 cells, was elevated in all experimental groups. Th1 activated mononuclear phagocytes (MP) as well as produced IL-12 to promote Th1-type cellular immune responses (52). IL-12, a polarizing cytokine, induces CD4 T cells to differentiate into a Th1 subset by two-stage differentiation a) IL-12 induces the outgrowth of IFN-γ Th1 cells, b) about half of Th1 cells ultimately gain CX3CR1 expression and exhibit a cytotoxic effector profile depending on the transcription factor ZEB2, which, in turn, produces IL-2 and IFN-γ (53, 54). IFN-γ-dependent macrophage activation is important for bactericidal functions. In our study, the IL-12 level in the IM group was dramatically higher than all other groups, and the highest protection rate provided by the IM group may, at least partly, depend on this. It has been shown that IL-12 can be used as an adjuvant to improve the effect of tuberculosis treatment (55) IL-6 has been reported to induce B cells to differentiate into and enhance CD8+ T cells differentiation. Perhaps dependent on the significant increase in IL-6 levels, only significant differences in CD8 levels were detected in the IM group in this study. IL-2 and IL-6 have regulatory effects on the differentiation of plasma cells and B cells, respectively, implying that increased levels of both have an important role in the regulation of humoral immunity (56).

Pathogenic *E. coli* CE9 could cause death in mice, accompanied by blood in the stool and urine. So the lungs, intestines, and kidneys were observed pathologically after the attack. The differences in pathological changes in the three organs mentioned above were obvious. The mice in the PBS group had severe lung, intestine, and kidney damage. The survivors in the traditional inactivated vaccine group were saved from death, but they still had pathological changes in several organs. We were surprised to find that the O group seemed to have better intestinal protection than the lungs and kidneys after the attack. This may be due to the local immunity of oral administration in the intestine. The three organs in the IM were essentially identical to those in the physiological states. This may be due to the fact that the IM route stimulated cellular immunity and high levels of humoral immunity. Specific IgG antibodies prevented bacterial infection through humoral immunity, and increased levels of IgA prevented bacterial adhesion and colonization. The high levels of Th1 and Th2 cytokines secretion also indicated that cellular immunity was activated, clearing pathogenic bacteria and protecting organs from infection. IL-12 has been shown to promote cytotoxicity of pulmonary mucosal-associated invariant T cells and improve clearance of pathogenic bacteria by mucosal immunity (57). Overall, high survival rates are generally associated with better tissue protection. It was reasonable to speculate that this enhancement of organ protection would be important for the health status and productive performance of surviving animals.

## Materials and methods

### Bacterial strain and plasmid

Bovine pathogenic *E. coli* isolates were identified as *E. coli* by streaking on McConkey medium and Eosin Methylene Blue Agar (EMB) medium. To further determine the genus of strain, the isolates were sent to Beijing Tsingke Biotechnology for 16srRNA sequencing. *Staphylococcus aureus* ATCC 25923 was purchased from the Institute of Microbiology, Chinese Academy of Sciences, and chemically competent DH5α cells were purchased from Shanghai Angyu Biotechnology. ΦX174 phage genomic DNA and pBV220 plasmid were maintained in our lab.

### Construction of lysis plasmid

The primer sequence were designed based on the *E* gene sequence of phage ΦX174 (accession no.: 9626372) and the sequence of Staphylococcus aureus nuclease A (accession no.: 21281729) published in Genebank. Homologous arms are bolded, and the 15bp flexible linker sequence underlined. Primers are listed in **Table 1** (**Table 1**). Staphylococcus aureus ATCC25923 was incubated at 37°C and 220rpm for 12h before being harvested as the template for PCR reaction. PCR conditions for production of amplicons for *E*-aa and *SNA* were as follows: bacterial template or Φ-X174 phage genomic DNA 2ul, EasyTaq DNA Polymerase (Transegen, China) 25ul, upstream primer 2ul, downstream primer 2ul, and ddH_2_O 19ul. PCR reactions were performed with a standardized cycling protocol: 94°C(1min 30s for DNA template, 5 min for colony PCR); 30 cycles of 94°C 30s, 60°C 20s, 72°C 30s; 72°C 5 min, 4°C hold. PCR products were analyzed by electrophoresis on 3% agarose gel using DL2000 (Takarabiomed, Japan) as marker. Target fragments were purified using the TIANgel Midi Purification Kit (TIANEN, China) following the instructions. Purified PCR products were sequenced *via* Sanger sequencing, and then stored at -20°C. Plasmid pBV220 was digested with *EcoRⅠ* and *BamHⅠ*, and the linear plasmid was harvested for further homologous recombination. Purified *E*-aa and *SNA* fragments were cloned into the linear pBV220 vector using the In-Fusion^®^ HD Cloning Kit (Takarabiomed, Japan).

**Table 1:** Primer sequences of gene *E* and *SNA*.

Gene	Sequence(5’～3’)	Product size(bp)
E	F:**TAAAAATTAAGGAGGGAATT**ATGGTACGCTGGACTTTGTGG	322
	R:TTGAAGTTGCAGAGCCACCGCCACC
SNA	F:**CGGTGGCTCT**GCAACTTCAACTAAAAAATTACATAAAGAACCTGC	481
	R:**TGGCTGCAGGTCGACGGATCC**TTATTGACCTGAATCAGCGTTGTC
IL-1β	F:AGCTCATATGGGTCCGACAG	174
	R:GGATGAGGACATGAGCACCT
IL-2	F:TGAGCAGGATGGAGAATTACAGG	120
	R:GTCCAAGTTCATCTTCTAGGCAC
IL-4	F:CTCATGGAGCTGCAGAGACTCTT	70
	R:CATTCATGGTGCAGCTTATCGA
IL-6	F:TGGAAATGAGAAAAGAGTTGTGC	121
	R:CCAGTTTGGTAGCATCCATCA
IL-12	F: GAGGCCTGTTTACCATTGGA	130
	R:TACTAAGGCACAGGGCCATC
IFN-γ	F:CTCAAGTGGCATAGATGTGGAAG	251
	R:TGACCTCAAACTTGGCAATACTC

The reaction mixture were added into competent *E. coli* DH5α cells and then incubated on ice for 30 min, followed by a 42°C heat shock for 90 s, and then another 2 min on ice. 500 μL of LB culture medium was added and incubated at 28°C and 220 rpm for 1 h. Bacterial cultures were inoculated into LB plates containing 50 ug/ml ampicillin and grown overnight at 28°C. Positive clones verified with PCR were sent for sequencing. Then, plasmid was extracted from the positive clones using the TIANprep Midi Plasmid Kit (Tiangen, China) and stored at -20°C for the next steps.

### Preparation of wild-type *E.coli* CE9 ghosts

*E. coli* CE9 was made chemically competent by CaCl_2_ treatment. Strain CE9 were grown in LB broth overnight, then diluted 1∶50 in fresh LB and incubated at 37°C until OD_600_ reached 0.3~0.5. The CE9 culture was transferred to a pre-chilled tube followed by ice incubation for 30 min. Cells were harvested by centrifugation at 4000 rpm at 4°C. After discarding the supernatant, the cells were gently suspended with a cold 0.1 mol/L CaCl_2_, centrifuged again, and then the culture was resuspended with 15% glycerol CaCl_2_ solution. All the samples were divided into 100 ul per tube and stored at -80°C.

Lysis plasmid pBV220-*E*-aa-*SNA* was transformed into CE9 chemically competent cells. The positive clones verified with PCR were named the CE9 ghost strain. Two milliliters of the overnight CE9 ghost cultures were added to the 100 ml LB medium. When cultures were grown to the OD_600_ reached 0.4~0.6 at 28°C, the temperature was increased rapidly to 42°Cto induce expression of the lysis gene. From then on, the OD_600_ of the bacterial cultures was measured every half hour until the OD_600_ no longer decreased. After expression, the cultures were centrifuged at 4°C, 8000 rpm for 20 min, washed three times with PBS, and divided into glass vials. To facilitate the storage and inactivation of live bacteria, BGs were frozen and lyophilized for 24h using BioCool lyophilizer FD-1-50. DNA extraction and colony counting were performed before and after expression. Counting colonies requires incubation at 28°C. Lysis efficiency was calculated using the equation: cleavage rate = (1- the colony count after the induction/colony count before induction) 100%. The BGs pellets were fixed with glutaraldehyde fixation buffer and then sent to the Large Scale Instrument and Equipment Sharing Service Platform of NEAU for inspection by TEM.

### Sterility and safety testing

An appropriate amount of the prepared CE9 ghost vaccine **resuspended** in sterile saline was spread evenly on the LB medium. The plates were incubated at 28 °C for 24 h, and then each well was examined for bacterial growth. For the safety testing, 15 female BALB/c mice (5-week-old) were divided into 5 groups, with 3 mice per group. The first group (control) was injected with PBS. The second group was injected intramuscularly with inactivated vaccines containing aluminum adjuvants. The others were administered with CE9 ghost vaccine at 10^10^ per mouse by oral, intramuscular, and subcutaneous routes, respectively. Mice were monitored continuously for 7 days.

### Preparation of inactivated vaccine containing aluminum adjuvants

*E. coli* CE9 strain stored at -80°C in 12.5% glycerol was inoculated with 5 mL of LB medium and incubated at 37°C for 12 h. The culture was inoculated 1:50 with 1000 mL of fresh LB medium and incubated at 37°C for 12 h. Cultures were diluted in sterile physiological saline and spread on LB agar plates. The colonies were counted after 24 h of incubation at 37 °C. The remaining bacterial culture was inactivated by adding formaldehyde at a ratio of 1:50 for 24 h. The inactivated bacterial culture was applied to the NB medium to check for the presence of viable bacteria. If not, samples were centrifuged at 4,000 rpm for 30 min, and precipitates were collected. The precipitate was resuspended with 50 mL of aluminum hydroxide (contains an aqueous solution of aluminum hydroxide (40mg/mL) and magnesium hydroxide (40mg/mL) plus inactive stabilizers)and physiological saline mixture was added, and finally sterile saline was added to fix the volume to the appropriate dilution for later immunization.

### Vaccination and challenge scheme

One hundred female 5-week-old BALB/c mice (RRID: IMSR_APB:4790) were randomly divided into 5 groups of 20 mice each, and fasted for 12 h before immunization. In group O, mice were water fasted for 8 h before immunization and given orally 100 μL of 5% NaHCO_3_ solution 30 min before immunization to neutralize stomach acid. Approximately 2 weeks after the primary immunization, the mice were given a booster immunization. The immunization dose for all groups was 1×10^9^ (100ul) per mouse. The mice were challenged with 5.5×10^8^ CFU wild-type *E. coli* CE9 by intraperitoneal injection on day 28 after the initial immunization. From the challenge on, the mice were monitored daily for 7 days with free access to food and water.

### Preparation of antigens and assessment of humoral responses

Blood samples and intestinal lavage fluid were collected before immunization at 7, 14, 21, and 28 days after the initial immunization. Blood samples were allowed to sit at room temperature for 1h at 4°C overnight until serum was collected. Serum samples and intestinal lavage fluid were stored at -80°C until assayed for specific antibodies. For the preparation of bacterial antigens, CE9 ghosts were resuspended in PBS with a 1.5h ultrasonic treatment. The protein concentration of the antigen sample was determined by the BCA method using TaKaRa BCA Protein Assay Kit (Takarabiomed, Japan). Antigen-specific antibodies were detected using indirect ELISA. Indirect ELISA experiments were performed as follows: the antigens were coated onto Costar 9018 enzyme plates with 42ug per well and incubated overnight at 4°C. The plate was washed with PBST for 300 μL per well for 5 min ×3 times. The blocking solution, 5% skim milk, was added to the plate at 200 ul per well and incubated for 2h at 37°C. Serum samples diluted in 1:19200 (intestinal lavage fluid diluted in 1:40) with PBS were added to each well in 100 μL and incubated at 37°C for 2 h. Horseradish peroxidase-labeled goat anti-mouse IgG (ZSGB-Bio Cat# ZB-2305, RRID : AB_2747415) or IgA (SouthernBiotech Cat# 1040-05, RRID : AB_2714213) diluted with PBST at 1:4000 was added and incubated at 37°C for 2 h. After three washes of PBST, TMB (Solarbio, China) was added and incubated for 10 min at room temperature, protected from light. Finally, 100 μL of 2 mol/L H_2_SO_4_ solution was added to each well to terminate the color development. Analysis of the level of specific antibodies induced by immunization was based on the OD_450_ values measured by using a microplate reader (BIO-TEK, USA).

### Assessment of cell-mediated immune responses by flow cytometry

At 28 days after the first immunization, the mouse spleen was aseptically harvested and ground in a 40um filter with an appropriate amount of PBS to isolate spleen mononuclear cells. Red Blood Cell lysis buffer (Solarbio, China) was used to resuspend the isolated cells, incubated at room temperature for 5 min, and then cell staining buffer (PBS with 1% BSA) was added and centrifuged at 1000 rpm for 5 min. The cells were washed one more time, resuspended with cell staining buffer, and the cell concentration was adjusted to 1×10^7^ CFU/ml. Anti-CD3-APC, anti-CD4-FITC, and anti-CD8-PE fluorescent antibodies (Elabscience, China) were added to 100 ul of cell suspension containing 1×10^6^ mononuclear cells and incubated for 30 min at 4°C. After the staining, 5ml of cell staining buffer was added and centrifuged. Finally, cells were resuspended with 500 ul of cell staining buffer, protected from light at 4°C until later assayed. Flow cytometry was performed (10,000 events) using BDAria (BD Biosciences, USA).

### Assessment of Th1/Th2 cytokines by RT-qPCR

On the 28th day after immunization, mice were anesthetized and peripheral blood was collected. Blood sample RNA was extracted using a blood RNA extraction kit (Invitrogen, USA), followed by a reverse transcription reaction. Cytokine levels were detected by RT-qPCR and quantified by the 2-ΔΔCt method using GAPDH as an internal reference gene. Results were normalized to the control group to calculate relative abundance. All RT-qPCR primers are provided in **Table 1**. The RT-qPCR was performed in a total volume of 20 μl containing cDNA (2 μl, 1:5 in ddH2O), SYBRs Premix Ex TaqTM reagents (10ul,Takarabiomed, Japan), primers (0.4 μM×2) and ddH2O (6.4ul). RT-qPCR conditions included a pre-incubation step (95°C for 30s), 40 cycles of amplification (95°C for 5s, 60°C for 30s) and a melting curve (95°Cfor 5s, 65°C for 1 min, then heat up to 95°C at 0.11°C per second, then cool down to 50°C 30s).

### Histopathology

Following bacterial challenge, the survivors were anesthetized to harvest their lungs, small intestines, and kidneys fixed in 4% paraformaldehyde. After rinsing with water, dehydration with gradient ethanol, and transparency with xylene, the tissues were paraffin-embedded in order to make paraffin blocks. Paraffin sections were sectioned at 3-5 um thickness and spread with slides. After dewaxing, hydration, hematoxylin and eosin staining was performed. The stained sections were dehydrated, hyalinized, and permanently mounted for observation.

## Data availability statement

The original contributions presented in the study are included in the article/supplementary material. Further inquiries can be directed to the corresponding authors.

## Ethics statement

The animal study was reviewed and approved by Laboratory Animal Ethics Committee of Northeast Agricultural University (SRM-11).

## Author contributions

JM designed and performed all the study. LL and YZ contributed to the experimental work. DL and JL contributed to the BGs vaccine and ELISA assays. JM and YF wrote the manuscript. YL and GW did corrections and critical review of this paper. All authors contributed to the article and approved the submitted version.

## Funding

This research was funded by National Key Research and Development Program of China (2016YFD0501310).

## Acknowledgments

The authors are thankful to Dongfang Shi at the College of Veterinary Medicine of Northeast Agricultural University for providing partial experimental equipment. Also, thanks to ZY and ZBB for their meaningful suggestions on the experimental design.

## Conflict of interest

The authors declare that the research was conducted in the absence of any commercial or financial relationships that could be construed as a potential conflict of interest.

## Publisher’s note

All claims expressed in this article are solely those of the authors and do not necessarily represent those of their affiliated organizations, or those of the publisher, the editors and the reviewers. Any product that may be evaluated in this article, or claim that may be made by its manufacturer, is not guaranteed or endorsed by the publisher.
